# Health professional's willingness to advocate for strengthening global commitments to the Paris climate agreement: Findings from a multi-nation survey

**DOI:** 10.1016/j.joclim.2021.100016

**Published:** 2021-05

**Authors:** Hye-ryeon Lee, Ian Pagano, Amanda Borth, Eryn Campbell, Benjamin Hubbert, John Kotcher, Edward Maibach

**Affiliations:** a University of Hawaii at Manoa, Honolulu, HI, USA; b George Mason University, Fairfax, VA, USA

**Keywords:** Climate change, Climate advocacy, Health professionals, Health communication, Paris agreement

## Abstract

Health professionals have the potential to address the health threats posed by climate change in many ways. This study sought to understand the factors that influence health professionals’ willingness to engage in climate advocacy. We hypothesized and tested a model with six antecedent factors predicting willingness to engage in advocacy for strengthening global commitments to the Paris Agreement. Using survey data from members of health professional associations in 12 nations (*n* = 3,977), we tested the hypothesized relationships with structural equation modeling. All of the hypothesized relationships were confirmed. Specifically, higher rates of perceived expert consensus about human-caused climate change predicted greater climate change belief certainty and belief in human causation. In turn, all three of these factors, including higher levels of perceived health harms from climate change, positively predicted affective involvement with the issue. Affective involvement positively predicted the feeling that health professionals have a responsibility to deal with climate change. Lastly, this sense that climate advocacy is a responsibility of health professionals strongly predicted willingness to advocate. As a unique study of predictors of health professionals’ willingness to advocate for climate change, our findings provide unique insight into how an influential set of trusted voices might be activated to address what is arguably the world's most pressing public health threat. Limitations of the study and suggestions for future research are presented, and implications for message development are discussed.

## Highlights

• Health professionals’ perception of scientific consensus on climate change and beliefs that human-caused climate change is indeed happening increase their involvement in the issue.
• An increase in affective issue involvement strongly influences the feeling that health professionals are responsible for climate advocacy.
• Feeling that health professionals are responsible for climate advocacy is strongly associated with increased willingness to participate in climate policy advocacy.
• The perception that climate change poses serious health threats increases affective involvement, appearing to indirectly increase willingness to advocate among health professionals.

## 1 Introduction

The health impacts of climate change are varied and far-reaching [1]. Certain communities and populations, including low-income and other marginalized communities, children, the elderly, and people with chronic conditions, are typically harmed first and worst [1]. To reduce the risks of health harms and protect human health, urgent climate actions are imperative. The current level of climate actions worldwide fall short of what is required to limit global warming to no more than 2 °C, the goal as ratified by all nations in the Paris Climate Agreement [1].

Health professionals can—and some argue must—play various roles in addressing climate change, including advocating for climate policy solutions [2], [3], [4]. Indeed, there is a long tradition of medical, nursing, and public health leadership in confronting large-scale health challenges [2,5,6]. Although many health professionals view climate change as a significant cause of health harm and feel a responsibility to educate the public and policymakers [7,8], anecdotal evidence suggests participation in advocacy efforts is less widespread. A descriptive report we published previously based on the same multi-nation survey showed that many health professionals cite a range of barriers that inhibit their participation in climate advocacy (e.g., lack of time and knowledge, belief that it won't make a difference, the topic is too controversial) [7]. To our knowledge, however, no research has been done to systematically examine the detailed relationships among the broader range of factors that might influence willingness to engage in climate advocacy as health issue. In this study, we aim to address this important gap in the literature.

Using survey data collected from health professionals in 12 different countries, we investigate key factors that are associated with willingness for climate advocacy, specifically willingness to participate in a global advocacy campaign by health professionals to encourage world leaders to strengthen their commitments to the Paris Agreement. Strengthening and achieving the goal of the Paris Agreement is urgent and critically important to global health, and active and broad-based involvement by health professionals in such an advocacy campaign will be an integral part of global efforts to reverse the harm of climate change. Better understanding of the factors that affect willingness for climate advocacy then will inform those who want to develop a campaign for climate advocacy in the future.

### 1.1 Predictors for willingness to advocate for climate action

Prior research has identified factors that influence people's decision to engage in civic or political action, including demographic factors, socialization or life experience factors, attitudinal factors, motivational factors, and mobilization factors (e.g., opportunity to participate) [9], [10], [11]. Our study population, health professionals, are highly educated and more likely to be engaged with important social issues than the general public. Thus, for our study population, attitudinal and motivational factors may play a more important role than demographic or socialization factors in the decision to participate in advocacy.

The term advocacy is defined in the dictionary as “the act or process of supporting a cause or proposal” [12]; however, the term is often associated with different types of actions [13]. Our study focuses on the act of participating in a global campaign by health professionals to encourage world leaders to strengthen their commitment to achieving the goals of the Paris Agreement. The essence of this action is one's willingness to publicly assert their support; we seek to understand the factors that influence whether a health professional is willing to engage in such an action.

Drawing on existing literature, we identified factors that may influence willingness to engage in climate advocacy: three fundamental beliefs about climate change; perceived health threats of climate change; affective issue involvement; and perceptions that climate advocacy is the responsibility of health professionals. We propose a theoretical model of health professionals’ willingness to advocate for climate change that specifies the relationships among these factors.

#### 1.1.1 Basic beliefs about climate change

Studies of the general public have identified several basic beliefs about climate change that influence people's understanding of the issue and support for climate action. The most obvious of these beliefs are (a) climate change is real and (b) human-caused [14]. In turn, these basic beliefs influence other factors such as perceived issue seriousness [15], affective issue involvement [14,16], support for national mitigation action and policies [17,18], and political activism [16].

The gateway belief model (GBM) posits perceived scientific consensus—the extent to which people think climate experts are convinced that human-caused climate change is occurring—as the most fundamental climate change belief [19]. Further, climate change is a complex, scientific phenomenon that most people do not understand well. The GBM proposes that perceived scientific consensus acts as a “gateway” to other cognitive and affective judgments. This assertion is supported by substantial empirical evidence, including experimental studies that show that highlighting the high level of scientific consensus leads people to update their beliefs about the consensus: Highlighting the high level of scientific consensus around climate change, in turn, strengthens people's belief that climate change is happening and human-caused and increases concern about climate change. These shifts in understanding lead people to become more supportive of policies to address the issue [18,19].

**Fig. 1:**
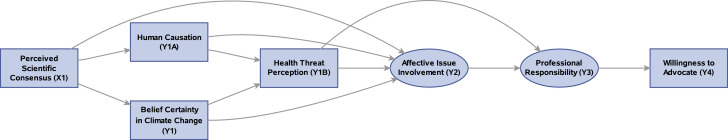
Hypothesized Model.

Thus, we hypothesize that health professionals’ perceptions of the scientific consensus influences their belief that climate change is real and human-caused (Fig. 1). Several studies have shown that these latter two beliefs are linked to perceived risks of climate change [20,21], therefore we hypothesize that health professionals’ beliefs in the reality of human-caused climate change positively influences their perceptions that climate change is a threat to health. Further, we hypothesize that all four of these beliefs positively influence health professionals’ affective involvement with the issue of climate change (i.e., the extent to which they see the issue as personally relevant and worry about it).

#### 1.1.2 Health threat perception

According to the Health Belief Model, risk or threat perception is an important factor that influences people's protective actions [22]. In the context of climate change, a range of harmful health effects have been identified [2], although their presence and severity vary by geographic location and its associated climatic conditions. Moreover, health professionals are not all equally well-informed about these risks [7]. Regardless of the reason for the variability, we expect that health professionals’ perceptions of serious health consequences due to climate change will predict both their affective issue involvement and their feelings of responsibility to take action to avert climate change.

#### 1.1.3 Affective issue involvement

Affective involvement—believing an issue has personal importance and being emotionally engaged with it—has been shown to increase people's attention to an issue, willingness to express an opinion, political participation, and opinion leadership [10,[23], [24], [25]]. In the context of climate change, issue involvement has been shown to influence both political and consumer advocacy and consumer behavior [14,16]. Based on this literature, we hypothesize that health professionals’ affective issue involvement will be positively related to their willingness to advocate for climate change.

#### 1.1.4 Perceived responsibility for advocacy

Felt responsibility, also known as personal responsibility, refers to the “extent to which individuals feel capable of and compelled to take useful action toward a desired result” [26]. The value-belief-norm theory (VBN) proposes that the feeling of personal responsibility is a key translator between abstract ideas and palpable actions [26], [27], [28]. Others have posited that worry about climate change increases feelings of personal responsibility, which in turn augments specific and personal climate actions [28]. Another study demonstrated that felt responsibility for adaptation led to taking adaptation actions, while felt responsibility for mitigation led to taking both mitigation and adaptation actions [26]. Thus, we hypothesize that the relationship between affective issue involvement and willingness to advocate for climate action will be mediated by the perception that health professionals have a responsibility to bring the health effects of climate change to the attention of the public and the policy makers.

## 2 Materials and method

### 2.1 Data and sample description

We surveyed the members of twelve medical and nursing professional societies in different countries to evaluate their views about climate change as a human health issue. The surveys were conducted online from October through December 2020 in collaboration with participating health professional associations. In total, 3977 health professionals completed the survey. The average participation rate was 10%; however, participation varied considerably from one society to another (reported in Appendix A along with detailed description of survey protocol). Men and women participated in the survey in roughly equal numbers (50.3% female, 47.4% male). The age of participants ranged from 19 to 109, with an average of 51 years. Most of the participants were physicians (94.8%). We have reported detailed descriptive data from the survey previously [7]. In this study, we focus on examining the hypothesized relationships between the key factors discussed above and health professionals’ willingness to participate in climate advocacy as health issue.

### 2.2 Measures

Measure details are reported below. Table 1 provides means, standard deviations, ranges, and correlations. Complete wording for questions used for the measures are provided in Appendix B.

**Table 1:** Descriptive Statistics and Correlation Matrix.

Measure	Item	N	Mean	SD	Range	X1	Y1	Y1A	Y1B	Y2_1	Y2_2	Y3_1	Y3_2	Y3_3	Y3_4	Y3_5	Y3_6	Y4
Perceived Scientific Consensus	X1	3891	87.7	15.4	0–100	1.00												
Belief Certainty in Climate Change	Y1	4527	8.32	1.27	1–9	0.47	1.00											
Human Causation	Y1A	4502	4.96	0.82	1–6	0.49	0.62	1.00										
Local Health Consequences	Y1B	4320	6.92	3.23	0–13	0.05	0.30	0.27	1.00									
Affective Issue Involvement	Y2_1	4459	3.49	0.75	1–4	0.39	0.62	0.62	0.40	1.00								
	Y2_2	4450	3.24	0.74	1–4	0.41	0.62	0.60	0.39	0.78	1.00							
Professional Responsibility	Y3_1	4101	4.32	0.99	1–5	0.26	0.54	0.47	0.41	0.62	0.60	1.00						
α = 0.92 (item 4 excluded)	Y3_2	4096	4.47	0.94	1–5	0.29	0.54	0.48	0.35	0.61	0.58	0.80	1.00					
	Y3_3	4085	4.35	0.99	1–5	0.28	0.46	0.44	0.31	0.51	0.52	0.59	0.59	1.00				
	Y3_4	1512	3.95	1.19	1–5	0.20	0.41	0.43	0.22	0.52	0.52	0.49	0.49	0.61	1.00			
	Y3_5	4077	4.51	1.03	1–5	0.36	0.63	0.57	0.39	0.69	0.65	0.71	0.73	0.64	0.59	1.00		
	Y3_6	4077	4.49	1.01	1–5	0.36	0.62	0.55	0.39	0.68	0.64	0.71	0.72	0.62	0.56	0.93	1.00	
Willingness to Advocate	Y4	4057	2.81	0.91	1–4	0.25	0.45	0.45	0.35	0.55	0.58	0.55	0.52	0.46	0.46	0.58	0.57	1.00

#### 2.2.1 Dependent variable

*Willingness to Advocate (Y4)* was measured by asking, “Would you personally be willing to participate in a global advocacy campaign by health professionals to encourage all world leaders to strengthen their commitment to achieving the goal of the Paris Climate Agreement?” ‘Yes’ responses were scored 3; ‘Possibly, but I would need more information’ and ‘I would support such a campaign, but I could not personally participate’ responses were scored 2; and ‘No’ responses were scored 1. Providing their name and an email address to receive information about how to participate in a global advocacy campaign increased their score by 1. The final DV ranged from 1 to 4.

#### 2.2.2 Predictors

*Perceived Scientific Consensus (X1)* was measured by asking “To the best of your knowledge, what percentage of climate scientists think that human-caused climate change is happening?” The variable ranged from 0 to 100, “Don't know” responses were considered missing.

*Belief certainty in climate change (Y1)* was measured by combining three questions, “Do you think that climate change is happening?’, if yes, “how sure are you that climate change is happening?”, and if no, “how sure are you that climate change is not happening?” The resulting variable ranged from 1 to 9, with higher values indicating higher certainty that climate change is happening. “Don't know” responses were recoded as the midpoint.

*Belief in human causation (Y1A)* was measured by a single item, “Assuming climate change is happening, do you think it is caused: entirely by human activities, mostly by human activities, about equally by human activities and natural changes, mostly by natural changes, entirely by natural changes, or by none of the above since climate change isn't happening.” The variable ranged from 1 to 6, with higher values indicating belief in more human causation.

*Health Threat Perception (Y1B)* was measured by asking, “How much, if at all, has climate change already adversely affected these (13 specific) health issues in your country?” Examples of individual items include “Heat-related illnesses” and “Vector-borne infectious diseases.” Responses were rescaled (0–1) and summed to create a composite score which ranged between 0 and 13, with higher values indicating greater threat perception. “Don't know” responses were considered as missing.

*Affective Issue Involvement (Y2)* was measured by two questions**,** “How worried are you about climate change?” and “How important is the issue of climate change to you personally?” Responses were standardized (1–4), with higher values indicating greater issue involvement.

*Perceptions of Health Professional's Responsibility (Y3)* was assessed by five Likert scale questions. An example is “Health professionals have a responsibility to bring the health effects of climate change to the attention of the public.” Higher values indicated more feelings of responsibility.

### 2.3 Data analysis

We performed structural equation modeling (SEM) with maximum-likelihood parameter estimation to assess the overall goodness-of-fit and estimate the individual parameters of the hypothesized model. Latent constructs represented the measures with multiple indicators (Y2 and Y3). We constrained the latent variable error variances to one. Because Y2 had only two indicators, we constrained its paths to equality to avoid under-identification. The items for Y3 were written in pairs with similar wording, so we allowed correlated errors. Five fit indices are reported, and standard thresholds for good model fit are: chi-square (*p* > .05), the standardized root mean square residual (SRMR < 0.05), the adjusted goodness-of-fit index (AGFI > 0.90), the root mean square error approximation (RMSEA < 0.07) with 95% confidence limits, and the Bentler comparative fit index (CFI > 0.90). The CALIS procedure in the SAS 9.4 software (SAS Institute Inc., Cary, NC) performed the analyses.

## 3 Results

**Fig. 2:**
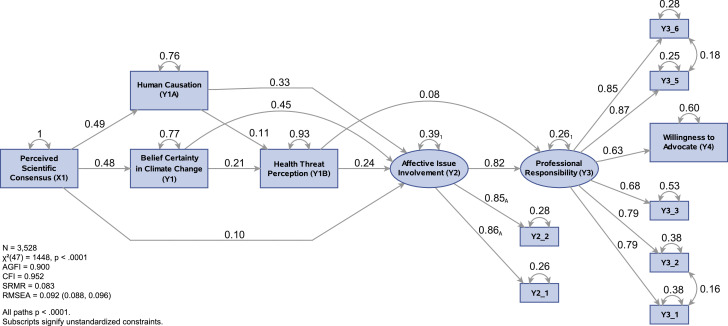
Hypothesized Model with Standardized Results.

**Fig. 3:**
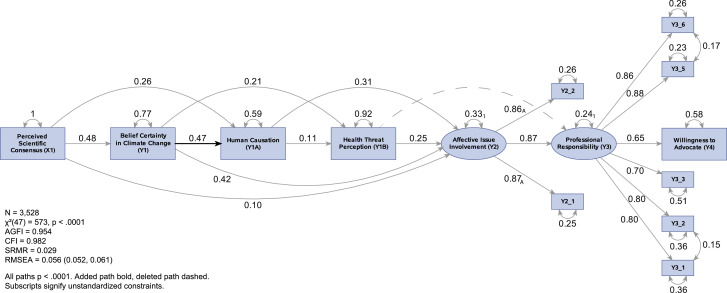
Revised Model with Standardized Results.

The results of the SEM for the hypothesized model are shown in Fig. 2. The model met the standard for acceptable goodness-of-fit based on two fit indices (AGFI = 0.90, CFI = 0.95), but failed to do so for the others. We examined the model's modification indices and created a revised model (Fig. 3). The revised model explains 42% of the variance in willingness to advocate, and all path coefficients are statistically significant (*p* < .0001). With the exception of the chi-square test, which is inflated by the large sample size, all fit indices were in the acceptable range (χ² = 573, p < .0001; SRMR = 0.03; AGFI = 0.95; RMSEA = 0.06; and CFI = 0.98). The revised model confirms all of the paths of the hypothesized model except for the path from climate change belief certainty to the belief in human causation, and the path from health threat perception to professional responsibility, which was removed due to low standardized value (*β < 0.01).*

Perceived scientific consensus is positively associated with climate change belief certainty (*β* = 0.48), and belief in human causation (*β* = 0.26). In turn, these three basic beliefs and perceived health threat are positively associated with affective issue involvement (*β* = 0.10, *β* = 0.42, *β* = 0.31, *β* = 0.25, respectively). Affective issue involvement has a strong positive association with the perception that climate advocacy is the responsibility of health professionals (*β* = 0.87), which in turn has a strong positive association with willingness to advocate (*β* = 0.65), as hypothesized.

## 4 Discussion

These results largely confirm our hypotheses and shed light on the factors that influence health professionals’ willingness to engage in at least one form of climate and health advocacy. In line with research literature on the GBM and our model, perceived scientific consensus and beliefs that human-caused climate change is indeed happening appear to increase affective involvement in the issue (i.e., the sense that climate change is personally important and worrisome). This increase in affective involvement appears to strongly influence the feeling that health professionals are responsible for climate advocacy, which is strongly associated with increased willingness to participate in climate policy advocacy. The perception that climate change poses serious health threats increases affective involvement, appearing to indirectly increase willingness to advocate.

Although we did not hypothesize a relationship between climate change belief certainty and belief in human causation, finding this relationship was not surprising given the high correlation between these two beliefs (*r* = 0.62). The only hypothesized relationship that was not supported by the data was the influence of health threat perception on feelings of professional responsibility, although the model did show an indirect influence.

These results also provide insights into how to design a campaign to engage health professionals in advocacy to strengthen the global commitment to the Paris Agreement. Our study suggests that promoting perceptions of proximal health harm, affective involvement in the issue, and feelings of professional responsibility for climate advocacy may be good foci for activating involvement in advocacy. Communicating about the extent of the scientific consensus of human-caused climate change may also be helpful.

Nearly three-quarters of our survey participants reported feeling a high degree of involvement with the issue [7] but translating this affective state into concrete behavioral involvement in advocacy may require some strong reinforcement. Modeling messages—in which opinion-leading health professionals demonstrate advocacy or talk about their approach to advocacy—can be an effective means of providing that reinforcement. Such messages may increase health professionals’ sense of self-efficacy as climate advocates, reinforce the notion that advocacy behavior by health professionals is normative, and demonstrate that taking actions consistent with one's values leads to experiencing positive emotion about oneself [28].

We found considerable variability in health threat perceptions across the thirteen health consequences specified in our study, some of which may be due to geographic variation. For instance, only 33% thought that water- and food-borne diseases were impacted by climate change compared to 65% for illness due to reduced outdoor air quality [7]. Using locally-relevant examples, educating health professionals about the full range of health impacts from climate change may increase their likelihood of engaging in climate advocacy.

Strengthening health professionals’ feelings of professional responsibility for climate advocacy is another promising strategy to encourage climate advocacy. While a large majority of study participants felt that health professionals are responsible to some degree for bringing the health effects of climate change to the attention of public and policy makers, only 26% were willing to participate in a global climate advocacy campaign. This gap between attitude and behavior may be reduced by strengthening feelings of personal and professional responsibility, increasing health professionals’ sense of self-efficacy to act effectively as advocates, and inviting them to engage in specific acts of advocacy [7,22,23]. Studies have shown that the most commonly cited reason for not engaging in climate advocacy is simply because people feel they have not been asked to get involved [29]. Clear messages that show how their perceptions of responsibility can be translated into action, as well as providing resources designed to enhance their sense of efficacy for participation, may be beneficial. In the long run, it may also be helpful to systematically integrate climate health and climate advocacy into health professional curricula, thus socializing future health professionals to see climate action as an essential element of their professional responsibility as reflected in Hippocratic Oath [30].

We note some limitations of the study. First, the data used in the study is cross-sectional in nature. Although we conducted SEM, the implied causal relationships should be interpreted with caution. Second, there are other potential predictors identified in the literature that were not included in our study. For example, self-efficacy has been shown to be an important predictor for activism for members of the general public [16] and may play an important role in health professionals' decision to engage in advocacy. Similarly, perceived barriers have been shown to play a role in people's decision to take action [22]. Future studies should include self-efficacy and key barrier measures in addition to the factors we studied. Third, our study focused on the specific action of joining a global climate advocacy campaign to support strengthening the Paris Agreement. There are many other important forms of climate health activism worthy of future study. Fourth, We note that the organizations that participated in the survey were a purposive sample chosen by leveraging existing connections through WHO, World Medical Association and Global Climate and Health Alliance. As such, they are not intended to be representative of health professional organizations at large globally, nor are they representative of all professional fields (e.g., nurses, social workers). A future study with more diverse organizations, both geographically and professionally, would broaden our understanding of the topic beyond what is reported in our study. Finally, our survey had a low response rate (10%). While our focus was on exploring theoretical relationships among variables rather than making population estimates, we note that our sample is likely not fully representative of the membership of each participating organization.

A stable climate is the most fundamental determinant of human health, and it is high time for comprehensive efforts to engage health professionals across the world to become vocal advocates for climate policy [3]. As the first study of detailed paths to climate advocacy among health professionals, this study adds valuable insights into the interrelationships among key predictors of willingness to advocate among this important population.

## Declaration of Competing Interest

Dr. John Kotcher reports a grant from World Health Organization.

## Appendix Supplementary materials

Image, application 1
