# Supplementary material for: Health professional's willingness to advocate for strengthening global commitments to the Paris climate agreement: Findings from a multi-nation survey
Source: J Clim Chang Health. 2021 May;2:None. doi: 10.1016/j.joclim.2021.100016 (PMC8262252; doi:10.1016/j.joclim.2021.100016)
Supplement: Supplementary file 1 [file mmc1.docx]

**Appendix A**

Survey Protocol, Timing, and Sample Size

Data was collected using the following basic protocol for each survey: Participating organizations emailed an initial endorsement letter from the professional organization's president or executive director inviting members to participate in the survey. Approximately 3 days later, members were emailed an invitation to take the survey online; the email included a URL link to the survey. Our survey platform (Qualtrics) prevents individuals from taking the survey more than once. For those who did not respond to the initial invitation, up to five reminders were sent over the following 4-5 weeks (spaced approximately one week apart) to increase participation.

Three versions of this basic protocol were administered, based on the requirements of the participating professional society. Version 1: Our research team conducted the survey using email addresses provided by the professional society. Version 2: The professional society conducted the survey on their own survey software platform using our survey questionnaire and protocol; in these cases, our research team conducted quality assurance prior to the survey launch to ensure that instrument was correctly programmed into their survey software. Version 3: The professional society distributed a URL link to the survey on our survey software platform using their own email distribution software rather than sharing member email addresses with us.

| Organization | Survey dates (mm/dd/yy) | Recruitment protocol | Total participants | Total population | Participation Rate |
| --- | --- | --- | --- | --- | --- |
| AGPJ - Association of General Practitioners of Jamaica | 10/09/20 to 11/17/20 | Version 3 | 10 | 110 | 9.1% |
| BMA - British Medical Association | 11/20/20 to 12/17/20 | Version 2 | 320 | 87924 | 0.4% |
| CMA - Canadian Medical Association | 10/09/20 to 10/25/20 | Version 2 | 2852 | 68398 | 4.2% |
| IAP - Indian Academy of Pediatrics | 10/09/20 to 11/17/20 | Version 1 | 385 | 9196 | 4.2% |
| KMA - Kuwait Medical Association | 10/09/20 to 11/17/20 | Version 3 | 78 | 1000 | 7.8% |
| NZNO - New Zealand Nursing Organization | 11/17/20 to 12/12/20 | Version 3 | 143 | 5000 | 2.9% |
| RACP - Royal Australasian College of Physicians (Australian Pediatric members) | 11/04/20 to 12/17/20 | Version 3 | 194 | 4930 | 3.9% |
| SAMA - South African Medical Association | 10/20/20 to 11/27/20 | Version 3 | 334 | 10564 | 3.2% |
| SOCHIMEF - Chilean Society of Family and Community Medicine | 11/11/20 to 12/09/20 | Version 1 | 83 | 395 | 21.0% |
| SOCMEF - Colombian Society of Family and Community Medicine | 11/17/20 to 12/15/20 | Version 1 | 29 | 129 | 22.5% |
| SUMEFAC - Uruguayan Society of Family and Community Medicine | 11/03/20 to 11/27/20 | Version 1 | 57 | 259 | 22.0% |
| WMA - World Medical Association (Associate members) | 10/09/20 to 11/17/20 | Version 3 | 169 | 900 | 18.8% |

**Appendix B**

Question Wording for Key Measures

**Dependent Variable: Willingness to Advocate (Y4)**

● Would you personally be willing to participate in a global advocacy campaign by health professionals to encourage all world leaders to strengthen their commitment to achieving the goal of the Paris Climate Agreement?

*Yes*

*Possibly, but I would need more information*

*I would support such a campaign, but I could not personally participate*

*No, and I would not support such a campaign*

● Please provide your name, country, and email address so that the Global Climate and Health Alliance can provide you with information about how you can support or participate in the global advocacy campaign, should you wish to.

**Predictors: Belief in Scientific Consensus (X1)**

To the best of your knowledge, what percentage of climate scientists think that human-caused climate change is happening? *(Yes, No, Don’t Know)*

**Predictors: Basic Belief that Climate Change is Happening (Y1)**

● Climate change refers to the idea that the world’s average temperature has been increasing for the past 50 to 100 years, may increase more in the future, and the world’s climate may be changing as a result. What do you think: Do you think that climate change is happening? *(Yes, No, Don’t Know)*

● How sure are you that climate change is happening? *(not at all, somewhat, very, extremely sure)*

● How sure are you that climate change is not happening? *(not at all, somewhat, very, extremely sure)*

**Predictors: Basic Belief that Climate Change is Caused by Humans (Y2A)**

● Assuming climate change is happening, do you think it is…

*Caused entirely by human activities*

*Caused mostly by human activities*

*Caused about equally by human activities and natural changes in the environment*

*Caused mostly by natural changes in the environment*

*Caused entirely by natural changes in the environment*

*None of the above because climate change isn’t happening*

**Predictors: Health Threat Perceptions (Y1B)**

● How much, if at all, has climate change already adversely affected these health issues in your country? (Not at all, Only a little, A moderate amount, A great deal)

1. Heat-related illnesses
2. Physical or mental harm from storms (including hurricanes) and floods
3. Physical or mental harm from forest fires or brush fires
4. Vector-borne infectious diseases
5. Water- and food-borne diseases
6. Anxiety, depression or other mental health conditions
7. Physical or mental harm from droughts
8. Illness due to reduced outdoor air quality (e.g., air pollution, pollen)
9. Loss of housing for residents displaced by extreme weather events
10. Disruptions to health care services for people with chronic conditions during extreme weather events
11. Hunger and malnutrition due to rising food prices
12. Increased poverty due to economic hardship, and resulting health problems
13. Violence, conflict, and/or resulting dislocation

**Predictors: Affective Issue Involvement (Y2)**

● How worried are you about climate change?

*Very worried*

*Somewhat worried*

*Not too worried*

*Not at all worried*

● How important is the issue of climate change to you personally?

*Not at all important*

*Not too important*

*Somewhat important*

*Very important*

*Extremely important*

**Predictors: Perceptions re Health Professionals Responsibility (Y3)**

● Please indicate the extent to which you agree or disagree with the following statements (5-point Likert scale)

1. Health professionals have a responsibility to bring the health effects of climate change to the attention of the public.
2. Health professionals have a responsibility to bring the health effects of climate change to the attention of policy makers.
3. My professional society should provide the opportunity for members to participate virtually (via the web) in meetings and conferences hosted by our society, so as to reduce emissions of climate pollutants from airplane or car travel.
4. Health professionals should actively encourage their nation's leaders to strengthen their nation's commitment to achieving the goal of the Paris Climate Agreement–which is to limit global warming to 2 degrees Celsius or less.
5. Health professionals should actively encourage all world leaders to strengthen all nations' commitments to achieving the goal of the Paris Climate Agreement–which is to limit global warming to 2 degrees Celsius or less.
